# Supplementary material for: Implementation of global health competencies: A scoping review on target audiences, levels, and pedagogy and assessment strategies
Source: PLoS One. 2020 Oct 1;15(10):e0239917. doi: 10.1371/journal.pone.0239917 (PMC7529249; doi:10.1371/journal.pone.0239917)
Supplement: S1 Appendix — (DOCX) [file pone.0239917.s002.docx]

**Appendix I: Search Terms by Database**

**Pubmed:**

("Global Health"[mh] OR "global health"[Mesh] OR "international health" OR "abroad" OR “one health” OR "public health”[Mesh])

AND/OR ("Education, Medical"[mh] OR "health education"[mh] OR "education" OR "preparation" OR "resources" OR "resource" OR "orientation" OR "training" OR "curriculum" OR "curricula" OR "teaching" OR "teach" OR "Program evaluation"[mh] OR "Students, Health Occupations"[mh] OR "student" OR "students" OR "trainee" OR "trainees") AND (“competenc*” OR “skill*” OR “outcome” OR “objective” OR “goal”) AND/OR (“level” OR “layer” OR “matrix” OR “ladder” OR “continuum” OR “milestone” AND/OR “assessment” OR “evaluation”)

**Embase:**

('global health'/exp OR 'global health' OR 'international health' OR 'abroad' OR 'international clinical' OR 'medical outreach') AND

('medical education'/exp OR 'health education'/exp OR 'education' OR 'preparation' OR 'resources' OR 'resource' OR 'orientation' OR 'training' OR 'curriculum' OR 'curricula' OR 'teaching' OR 'teach' OR 'program evaluation'/exp OR 'health students'/exp OR 'student' OR 'students' OR 'trainee' OR 'trainees') AND (‘competence’ OR ‘skill’ OR ‘outcome’ OR ‘objective’ OR ‘goal’) AND/OR (‘level’ OR ‘layer’ OR ‘matrix’ OR ‘ladder’ OR ‘continuum’ OR ‘milestone’ OR ‘assessment’ OR ‘evaluation’)

**ERIC:**

("Global Health" OR "international health" OR "public health”)

AND/OR ("education" OR "preparation" OR "resources" OR "resource" OR "orientation" OR "training" OR "curriculum" OR "curricula" OR "teaching" OR "teach" OR "student" OR "students" OR "trainee" OR "trainees") AND (“competence” OR “competency” OR “skill*” OR “outcome” OR “objective” OR “goal”) AND/OR (“level” OR “layer” OR “matrix” OR “ladder” OR “continuum” OR “milestone” OR “assessment” OR “evaluation”)

**Google Scholar and Google:**

We conducted systematic searches of combinations of the keywords in each of the four concepts of the search (Table 1). For example, Global Health AND education AND/OR competence AND/OR level.
