# Supplementary material for: Implementation of global health competencies: A scoping review on target audiences, levels, and pedagogy and assessment strategies
Source: PLoS One. 2020 Oct 1;15(10):e0239917. doi: 10.1371/journal.pone.0239917 (PMC7529249; doi:10.1371/journal.pone.0239917)
Supplement: Attachment — Submitted filename: PONE-D-19-35888_reviewer.pdf [file pone.0239917.s003.pdf]

PLOS ONE

Implementation of Global Health Competencies: A Scoping Review on Target Audiences, Levels, and Pedagogy and Assessment Strategies

--Manuscript Draft--

Manuscript Number:	PONE-D-19-35888
Article Type:	Research Article
Full Title:	Implementation of Global Health Competencies:<br>A Scoping Review on Target Audiences, Levels, and Pedagogy and Assessment Strategies
Short Title:	Implementation of Global Health Competencies
Corresponding Author:	Meike Schleiff<br>Johns Hopkins University Bloomberg School of Public Health<br>Baltimore, MD UNITED STATES
Keywords:	global public health; public health training programs; milestones; levels of achievement; pedagogy; assessment strategies; global public health workforce; LMIC; competency-based education; CBE; curricula; evaluation; target audiences
Abstract:	Background: As the field of global health expands the recognition of structured training for field-based public health professionals has grown. Substantial effort has gone towards defining competency domains for public health professionals working globally. However, there is limited literature on how to implement competency-based training into learning curricula and evaluation strategies.<br>Objectives: This scoping review seeks to collate the current status, degree of consensus, and best practices, as well as gaps and areas of divergence, related to the implementation of competencies in global public health curricula. Specifically, we sought to examine (i) the target audience, (ii) the levels or milestones, and (iii) the pedagogy and assessment approaches.<br>Sources of Evidence: A review of the published and grey literature was completed to identify published and grey literature sources that presented information on how to implement or support global health and public health competency-based education programs. In particular, we sought to capture any attempts to assign levels or milestones, any evaluation strategies, and the different pedagogical approaches.<br>Results: A total of 21 documents were included, of which 18 were peer-reviewed and three were from the grey literature. Most of the sources focused on post-graduate public health students, professional trainees pursuing continuing education training, and clinical and allied health professionals working in global health. Two approaches were identified to defining skill level or milestones, namely: (i) defining levels of increasing ability or (ii) changing roles across career stages. Pedagogical approaches featured field experience, direct engagement, group work, and self-reflection. Assessment approaches included self-assessment surveys as well as 360 evaluations.<br>Conclusions: The implementation of global health competencies needs to respond to the needs of specific agencies or particular groups of learners. A milestones approach may aide these efforts while also support monitoring and evaluation. Further development is needed to understand how to assess competencies in a consistent and relevant manner.
Order of Authors:	Meike Schleiff
	Bhakti Hansoti
	Anike Akridge
	Caroline Dolive
	David Hausner
	Anna Kalbarczyk
	George Pariyo
	Thomas Quinn
	Sharon Rudy

Powered by Editorial Manager® and ProduXion Manager® from Aries Systems Corporation

	Sara Bennett
**Additional Information:**	
**Question**	**Response**
**Financial Disclosure**<br><br>Enter a financial disclosure statement that describes the sources of funding for the work included in this submission. Review the submission guidelines for detailed requirements. View published research articles from *PLOS ONE* for specific examples.<br><br>This statement is required for submission and **will appear in the published article** if the submission is accepted. Please make sure it is accurate.<br><br>**Unfunded studies**<br>Enter: *The author(s) received no specific funding for this work.*<br><br>**Funded studies**<br>Enter a statement with the following details:<br>• Initials of the authors who received each award<br>• Grant numbers awarded to each author<br>• The full name of each funder<br>• URL of each funder website<br>• Did the sponsors or funders play any role in the study design, data collection and analysis, decision to publish, or preparation of the manuscript?<br>• **NO** - Include this sentence at the end of your statement: *The funders had no role in study design, data collection and analysis, decision to publish, or preparation of the manuscript.*<br>• **YES** - Specify the role(s) played.<br><br>* typeset	SB and TCQ<br>The STAR project is supported through Cooperative Agreement No. 7200AA18CA00001 by the United States Agency for International Development (USAID).<br>The funders had no role in study design, data collection and analysis, decision to publish, or preparation of the manuscript.
**Competing Interests**<br><br>Use the instructions below to enter a competing interest statement for this submission. On behalf of all authors, disclose any competing interests that could be perceived to bias this work—acknowledging all financial support and any other relevant financial or non-financial competing interests.	The authors have declared that no competing interests exist.

Powered by Editorial Manager® and ProduXion Manager® from Aries Systems Corporation

This statement **will appear in the published article** if the submission is accepted. Please make sure it is accurate. View published research articles from *PLOS ONE* for specific examples.<br><br>**NO authors have competing interests**<br><br>Enter: *The authors have declared that no competing interests exist.*<br><br>**Authors with competing interests**<br><br>Enter competing interest details beginning with this statement:<br><br>*I have read the journal's policy and the authors of this manuscript have the following competing interests: [insert competing interests here]*<br><br>* typeset	
**Ethics Statement**<br><br>Enter an ethics statement for this submission. This statement is required if the study involved:<br><br>• Human participants<br>• Human specimens or tissue<br>• Vertebrate animals or cephalopods<br>• Vertebrate embryos or tissues<br>• Field research<br><br>Write "N/A" if the submission does not require an ethics statement.<br><br>General guidance is provided below. Consult the submission guidelines for detailed instructions. **Make sure that all information entered here is included in the Methods section of the manuscript.**	N/A

**Format for specific study types**<br><br>**Human Subject Research (involving human participants and/or tissue)**<br>• Give the name of the institutional review board or ethics committee that approved the study<br>• Include the approval number and/or a statement indicating approval of this research<br>• Indicate the form of consent obtained (written/oral) or the reason that consent was not obtained (e.g. the data were analyzed anonymously)<br><br>**Animal Research (involving vertebrate animals, embryos or tissues)**<br>• Provide the name of the Institutional Animal Care and Use Committee (IACUC) or other relevant ethics board that reviewed the study protocol, and indicate whether they approved this research or granted a formal waiver of ethical approval<br>• Include an approval number if one was obtained<br>• If the study involved *non-human primates*, add *additional details* about animal welfare and steps taken to ameliorate suffering<br>• If anesthesia, euthanasia, or any kind of animal sacrifice is part of the study, include briefly which substances and/or methods were applied<br><br>**Field Research**<br><br>Include the following details if this study involves the collection of plant, animal, or other materials from a natural setting:<br>• Field permit number<br>• Name of the institution or relevant body that granted permission	
**Data Availability**<br><br>Authors are required to make all data underlying the findings described fully available, without restriction, and from the time of publication. PLOS allows rare exceptions to address legal and ethical concerns. See the PLOS Data Policy and FAQ for detailed information.	Yes - all data are fully available without restriction

A Data Availability Statement describing where the data can be found is required at submission. Your answers to this question constitute the Data Availability Statement and **will be published in the article**, if accepted.<br><br>**Important:** Stating 'data available on request from the author' is not sufficient. If your data are only available upon request, select 'No' for the first question and explain your exceptional situation in the text box.<br><br>Do the authors confirm that all data underlying the findings described in their manuscript are fully available without restriction?	
**Describe where the data may be found in full sentences. If you are copying our sample text, replace any instances of XXX with the appropriate details.**<br><br>• If the data are **held or will be held in a public repository**, include URLs, accession numbers or DOIs. If this information will only be available after acceptance, indicate this by ticking the box below. For example: *All XXX files are available from the XXX database (accession number(s) XXX, XXX.).*<br>• If the data are all contained **within the manuscript and/or Supporting Information files**, enter the following: *All relevant data are within the manuscript and its Supporting Information files.*<br>• If neither of these applies but you are able to provide **details of access elsewhere**, with or without limitations, please do so. For example:<br><br>*Data cannot be shared publicly because of [XXX]. Data are available from the XXX Institutional Data Access / Ethics Committee (contact via XXX) for researchers who meet the criteria for access to confidential data.*<br><br>*The data underlying the results presented in the study are available from (include the name of the third party*	All relevant data are within the manuscript and its Supporting Information files.

and contact information or URL).<br>• This text is appropriate if the data are owned by a third party and authors do not have permission to share the data.<br><br>* typeset	
Additional data availability information:	

Manuscript

Click here to access/download;Manuscript;Implementation of Global Health Competencies 2020 01 06.docx

Formatted

Implementation of Global Health Competencies:
A Scoping Review on Target Audiences, Levels, and Pedagogy and Assessment Strategies

Co-Authors:
- Meike Schleiff, DrPH, MSPH, Johns Hopkins School of Public Health, Baltimore, MD, USA
- Bhakti Hansoti, MBChB, MPH, PhD, Johns Hopkins School of Medicine, Baltimore, MD, USA
- Anike Akridge, MPH, Public Health Institute, Washington, DC, USA
- Caroline Dolive, MA, Public Health Institute, Washington, DC, USA
- David Hausner, PhD, MPH, MIA, Public Health Institute, Washington, DC, USA
- Anna Kalbarczyk, MPH, Johns Hopkins School of Public Health, Baltimore, MD, USA
- George Pariyo, PhD, Johns Hopkins School of Public Health, Baltimore, MD, USA
- Thomas C. Quinn, MD, MSc, Johns Hopkins School of Medicine, Baltimore, MD, USA
- Sharon Rudy, PhD, Public Health Institute, Washington, DC, USA
- Sara Bennett, PhD, MPhil, Johns Hopkins School of Public Health, Baltimore, MD, USA

Formatted: Font: Bold

Formatted: Font: Bold

Formatted: List Paragraph, Bulleted + Level: 1 + Aligned at: 0.25" + Indent at: 0.5"

Abstract

Background: As the field of global health expands the recognition of structured training for field-based public health professionals has grown. Substantial effort has gone towards defining competency domains for public health professionals working globally. However, there is limited literature on how to implement competency-based training into learning curricula and evaluation strategies.

Objectives: This scoping review seeks to collate the current status, degree of consensus, and best practices, as well as gaps and areas of divergence, related to the implementation of competencies in global public health curricula. Specifically, we sought to examine (i) the target audience, (ii) the levels or milestones, and (iii) the pedagogy and assessment approaches.

~~Methods~~Sources of Evidence: A review of the published and grey literature was completed to identify published and grey literature sources that presented information on how to implement or support global health and public health competency-based education programs. In particular, we sought to capture any attempts to assign levels or milestones, any evaluation strategies, and the different pedagogical approaches.

~~Findings~~Results: A total of 21 documents were included, of which 18 were peer-reviewed and three were from the grey literature. Most of the sources focused on post-graduate public health students, professional trainees pursuing continuing education training, and clinical and allied health professionals working in global health. Two approaches were identified to defining skill level or milestones, namely: (i) defining levels of increasing ability or (ii) changing roles across career stages. Pedagogical approaches featured field experience, direct engagement, group work, and self-reflection. Assessment approaches included self-assessment surveys as well as 360 evaluations.

Conclusions: The implementation of global health competencies needs to respond to the needs of specific agencies or particular groups of learners. A milestones approach may aide these efforts while also support monitoring and evaluation. Further development is needed to understand how to assess competencies in a consistent and relevant manner.

Introduction

Global health is defined by Koplan et al. (2009) as "an area for study, research, and practice that places a priority on improving health, and achieving equity in health for all people worldwide."(1) Global health addresses the root causes of disease through an interdisciplinary and population-based effort, as well as an individual care perspective. (1) In recent years, the role of global health professionals has evolved in response to the needs of an interconnected world, from a disease-oriented and predominantly curative focus towards promoting holistic interventions which more appropriately address sociocultural influences on health, promote equity, and respond to complex societal needs.(2) Global health professionals work across many contexts and include public health workers dealing with health and its related influences and determinants in low resource settings, as well as, those supporting policy-making, medical and treatment guidelines development, budgeting and financing, service provision, data management and use, training and capacity building, and other aspects of public health programming. Training of these diverse participants engaged in the field of global health needs to extend beyond disease-specific and other technical knowledge to include a skill base that matches the challenges of working in an interdisciplinary, cross-cultural, multi-sectoral environment to improve health outcomes worldwide.(3-6)

The demand and need to define success and guide successful training in global health has led many groups, institutions, and professional societies to define sets of global health competencies. Competency-based education aims to move away from traditional learning assessment approaches—such as counting hours spent learning—to capturing the "knowledge, skills, and attitudes [or abilities] required for an acceptable level of practice."(7) This approach opens up opportunities to focus on job performance, as well as allowing for the definition of

levels of skill for assessment of progress.(8, 9) Competency-based training has gained popularity in recent decades across many disciplines, including education, medicine, public health, and global health. (9-11)

In global health, agencies and consortia, such as the Association of Schools and Programs in Public Health (ASPPH), the Consortium of Universities for Global Health (CUGH), and Global Health Education Consortium (GHEC), have developed tailored sets of competencies to match their specific areas of expertise and target audiences of learners.(12-15) The CUGH competency set was developed based on a review of the existing literature and thirty professional society and organization websites. On the other hand, the ASPPH utilized a multi-stage Delphi process to develop their competency set.(14, 16) The United States Agency for International Development (USAID) and the Public Health Foundation (PHF) have developed tailored sets of competencies for the different public health workforce roles in their respective organizations.(4, 17-20) In an attempt to document the various approaches, a review of global health competencies published in 2017 examined 13 documents that included competency domains and proposed a set of competencies closely resembling the CUGH competency domains. This framework captures both the public health technical skills as well as "soft skills" / "leadership skills" that are applicable across the range of public health and global health roles.(7)

While broad consensus is being reached at the stage of defining competency domains for the fields of public health and global health, it is significantly more difficult to decipher is how training programs, institutions, and organizations are incorporating global health competencies into their learning and performance activities.(21) Many competency domains mention specific technical skills or knowledge areas, for example, within the domain of program management, competency is defined as the "ability to design, implement, and evaluate global health programs

to maximize contributions to effective policy, enhanced practice, and improved and sustainable health outcomes."(14) While such descriptions provide a broad overview of what each domain should contain, they are often too general to design learning activities and track achievement of specific standards or learning objectives by a learner. Furthermore, there is now an increasing desire to codify and track levels of achievement and quantify assessment of global health practice.(22)

Several groups have worked on developing structured assessment approaches across public health competencies. (23) In 2003, the Public Health Foundation's Council on Linkages Between Academia and Public Health Practice developed a three-tiered model to assess a range of public health skills.(19) Others have also developed surveys to assess communications, leadership, and analytical skills among public health professionals.(24) Another group developed a strategy to assess skills such as mobilizing partnerships and enforcing laws and regulations in public health nurses in Illinois.(25) Most recently, initiatives have begun to identify how these competencies can be applied to professionals in low and middle-income countries (LMICs). (26)

In this paper, we describe the current approaches to global public health competency-based education that has been developed and discuss the opportunities and needs for further development. Specifically, we sought to examine (i) the target audience, (ii) the levels or milestones, and (iii) the pedagogy and assessment approaches.

Materials and Methods

<u>Scope</u>

In this paper we focus on two professional fields; "Public Health" and "Global Healt[illegible] While both fields have different origins and emphases, they overlap in terms of training content and have overlapping competency needs, as well as a growing imperative to work closely and

seamlessly together in teams and across agencies. Further, individuals with both public and global health backgrounds may fill similar jobs and roles in many agencies, particularly in international organizations.(27-29) Lastly, professionals from both fields have been working to identify approaches to implement competency domains into their respective curricula.(7, 14, 30) Throughout this paper, we refer jointly to these fields as “Global Public Health," reflecting our focus on the professionals who are engaged in implementing public health programming globally.

Search Strategy

We searched PubMed, Embase, ERIC, and Google/Google Scholar for articles and documents published from 2003 through September 15, 2019. This timeframe was chosen because it was expected to capture the majority of the literature on competencies in public health and global health.(7) The search encompassed four concepts in total (Table 1), which included global health and public health, education/capacity building; competency; milestone or level. A scoping review methodology was employed given the broad nature of our search, and a lack of a focused question but rather a need to capture a breadth of knowledge.(31) We searched broadly for initiatives and studies looking at how public health and global health competency sets are being utilized and evaluated, including those from high-resource settings aimed at training professionals to work globally, including across low- and middle-income settings. Terms were identified inductively from the search results that could enable us to develop more targeted searches, and so we added terms to the search strategy (Appendix 1). We adapted the search strategy for each database to minimize the possibility of missing relevant materials. We also reviewed citations of the relevant documents that we identified.

Table 1: Concepts and specific terms utilized in the literature search

**Concept**	**Search Terms**

1. Global Health AND	global health OR international health OR one health OR public health
2. Education/capacity-building AND/OR	education* OR training* OR university* OR curriculum* OR college* OR capacity OR workshop OR mentor
3. Competency AND	Competency* OR skill* OR outcome* OR objective*
4. Milestone AND/OR assessment	level OR layer OR matrix OR ladder OR continuum OR milestone AND/OR assessment OR evaluation

Data ~~Extraction~~ Charting and Synthesis

All included articles were reviewed in their entirety to understand in-depth the experience and status of competency-based assessment approaches in the field of global health. We ~~attempted to~~ captured specific data on target audiences, models for defining skill level, pedagogy, and assessment approaches and prepared a matrix, which was then refined into Tables 2 and 3.

Results

The scoping review identified 68 documents that presented data on a competency-based training program for global public health professions, which were reviewed in their entirety. Of these, 21 documents are featured in this comprehensive review (Tables 2 & 3). Of these, 18 were peer-reviewed published articles and three were other documents that included reports, policy guidelines, and electronic versions of tools. The documents had publication dates spanning from 2011-2019; older materials that were identified in the search were either solely focused on US-based public health professionals or did not include any information on the implementation of competencies. We cited a number of these older documents in the introduction to this paper in order to provide some context and acknowledge prior work undertaken in this field.

Three themes were identified for exploration in this literature review (i) target audiences; (ii) milestones or levels; and (iii) pedagogical and assessment strategies and are presented sequentially in the remainder of the results section. These topics serve as the organizing framework for this section. Tables 2 and 3 provide an overview of the findings across each topic, organized chronologically by year of publication. Table 2 focuses on findings that are non-clinically oriented or multi-disciplinary programs, whereas Table 3 focuses on clinically oriented programs. We have separated these two categories in order to be able to compare across programs focusing on public health professionals versus those providing global health content to other health professionals, and the inherent nuances of training these two groups of professionals.

Table 2: Non-clinically Oriented Competency Frameworks Use and Target Audiences; Levels and Milestones; and Pedagogy and Assessments

Article/Report	Competency Framework	Target Audiences	Levels and Milestones	Pedagogy and Assessments
Cole *et al*. (2011)	CCPHC (Core Competencies of Public Health in Canada), CCGHR (Canadian Coalition for Global Health Research (32) (Canada)	Post-graduate students seeking careers in public health or global health	Proposes complementary milestones for global health practitioners planning to work in practice or research settings to achieve during post-graduate training	Recommended pedagogical approaches include active reflection, direct engagement with diverse stakeholders to analyze challenges, and seeking mentorship in areas of interest. No assessment tools developed or discussed.
Gruppen *et al*. (2012)	None cited.	Health professionals	Cites and illustrates Miller's Pyramid, which includes four levels from 1) knows, 2) knows how, 3) shows, to 4) does	Illustrates the diversity of pedagogical and assessment approaches that could be utilized depending on the competency.<br>• Pedagogy: emphasizes use of simulation, small group work, and self-directed exploration and application.<br>• Assessments include working with standardized patients, oral or written examples, and supervised practice.
Jogerst *et al.* (CUGH) (2015)	Developed own for this paper (USA-based)	Global health trainees from a variety of disciplines	• Level I. Global Citizen Level, focused on awareness of global health among post-secondary students<br>• Level II. Exploratory Level, focused on students considering a future in global health<br>• Level III. Basic Operational level, with two sub-levels differentiating between clinicians and discipline-specific professionals working in global health and those working on managing global health programs<br>• Level IV. Advanced: plans long-term engagement in global health with leadership positions	Mentions the need for further dialogue and work in this area.
Sharma, *et al*. (2013)	Public health Foundation (PHF)/Council on Linkages Between Academia and Public Health Practice (19)	MPH students	Relates to PHF levels in discussion; need for further research to map to these levels to specific competencies or milestones.	Mentions the need for further dialogue and work in this area.

Article/Report	Competency Framework	Target Audiences	Levels and Milestones	Pedagogy and Assessments
	(India)			
Afya Bora Fellowship (2013)	Developed own (33) (Sub-Saharan Africa)	Post-graduate health professionals planning to lead and manage programs in Africa	Utilizes four skill levels on a Likert scale from “weak” to “excellent”	Use of online modules (lectures, discussion) as well as in-person intensive, group-oriented sessions. Self-reported survey administered at the beginning and end of each module of the curriculum.
Winskell, *et al.* (2014)	ASPPH (34)(USA-based)	MPH students	Specific goals to be achieved by the completion of the MPH degree program	Use of case studies, proposal development, group discussion.
ASPPH (2016)	ASPPH (35) (USA-based)	MPH students	Specific milestones for completion of MPH are included across all competency domains.	Not discussed beyond standard approaches to graduate education including lectures, group work, and writing papers. No assessment tool developed, nor scale discussed.
USAID (2017)	USAID (18) (USA-based)	USAID employees	Three levels of public health competence: Basic, Intermediate, and Advanced are described and relate to kinds of roles held.	Provide online and in-person trainings as well as access to other professional development opportunities. Assessment in the form of evaluations of trainings as well as performance reviews and feedback from onsite managers.
Eichbaum (2017)	CUGH (14) and ACGME (36) (USA-based)	Students and trainees from high resource-settings working in low-resource contexts	Not discussed.	Advocates for differentiation between acquired knowledge and skills (individual) and participatory knowledge and skills (collective) in evaluation approaches. Recommends “self-directed assessment” to evaluate these by incorporating feedback from multiple sources, including faculty, health system, and self. No tool developed.
Sawleshwarkar & Negin (2017)	Defined set similar to CUGH (14) (USA-based)	Post-graduate public health students	Defined “key elements” in the form of knowledge/skills for each competency domain to be obtained by the end of a training program.	Not discussed explicitly; no tools developed or tested.
Hamer, *et al.* (2019)	Developed own (37)	Mentors in LMICs who are involved in global health research	Not mentioned separately from assessment approaches.	Assessment approaches included self-reporting, monitoring mentor products from research (proposals, publications, etc.), review of mentee products, mentee satisfaction and feedback, obtaining funding, and frequency of meetings with mentees.
Hobson, *et al.* (2019)	Council on Education for Public Health (CEPH)(38) (USA, Canada, Lebanon, Mexico, and West Indies)	MPH students	Specific qualifications for achievement of evaluation related CEPH competencies by the end of MPH degree program.	Pedagogy and assessment approaches included lectures, readings, paper-writing, group projects, field evaluations, and journaling.

Table 3: Clinically Oriented Competency Framework Use and Target Audiences, Levels and Milestones, and Pedagogy and Assessments

Article/Report	Competency Framework	Target Audiences	Levels and Milestones	Pedagogy and Assessments
Redwood-Campbell, *et at*. (2011)	CanMEDS (39) (Canada)	Medical students and residents planning careers in public health or global health	Not discussed beyond targets or skills to achieve by the end of the medical education program.	Advocates for evaluation of service learning, field placements utilizing self-reflection, group learning, simulation, and apprenticeship. No assessment tools developed or proposed.
Gladding, *et al.* (2012)	ACGME (36) (USA-based)	Pediatric residents participating in an international elective.	Not discussed beyond targets or skills to achieve by the end of the medical education program.	Reflective essays were utilized to qualitatively evaluate progress towards ACGME domains as well as clarify personal goals and values.
Veras, *et al.* (2013)	CanMEDS (40) (Canada)	Occupational therapy and physiotherapy students studying global health	None specifically mentioned; the study explored existing knowledge and skills as well as learning needs across competencies.	Online assessment survey included 3-point scale of global health knowledge, a 5-point scale of global health skills, and a 5-point scale of global health learning needs.
Munyewende, *et al*. (2016)	WHO (41) and ICN (International Council of Nurses) managerial competencies (42) (South Africa)	Clinic nursing managers working in public health programs in South Africa.	Not discussed beyond general reference to expected skill levels for nurse managers.	Manager self-assessment and assessment by subordinates. 360-degree competency evaluation tool developed with questions on a 10-point scale of increasing skill level.
Wroe, *et al*. (2017)	ASPPH (35) (USA-based)	Internal medicine residents participating in global health training	Not discussed.	Emphasis on opportunities to engage in real-life practice or simulations. Assessment instrument is a student interview-based tool (scenarios). Designed for use in evaluating portions of residency programs, job candidate evaluation, and ongoing practical trainings.
Knight, *et al*. (2017)	HPCSA (Health Professions Council of South Africa) (43) (South Africa)	Clinicians receiving public health training	Measured whether specific outcomes or skills were acquired or achieved by the end of the program.	Pedagogy includes field placements working on community diagnosis and planning and evaluating a program. Assessment was a student online survey covering specific skills within each competency domain. The survey used a Likert scale with agree/neither agree nor disagree/disagree options for whether learners has acquired that skill. It also allowed students to provide qualitative feedback on their experience.
Kim, *et al*. (2017)	None mentioned. (South Korea)	Graduate nursing students studying global health	Not discussed.	Advocates for going beyond didactic lessons and incorporating simulations and field-based scenarios. Assessment approach was the application of *Veras, et al. (2013)* tool.
Douglass, *et al.* (2017)	CUGH Expert Working Group (EWG) (14) (USA-based)	Emergency medicine residents studying global health	• Level 1: Focus on awareness and knowledge<br>• Level 2: Focus on understanding and describing	Suggests appropriate pedagogy and assessments for each level, with overlap between levels:<br>• Level 1: Group discussions, course assessments, simulations, essays

Article/Report	Competency Framework	Target Audiences	Levels and Milestones	Pedagogy and Assessments
			• Level 3: Focus on participation, observation, and application<br>• Level 4: Focus on collaboration, management, and evaluation<br>• Level 5: Focus on creating, advocacy, and leadership	• Level 2: Group discussions, simulations, observation<br>• Level 3: Self-assessments, assessments from field experiences (360 evaluations)<br>• Level 4: Mentor evaluations, presentations<br>• Level 5: Colleague or partner evaluations, academic productivity, or curriculum development
Kelly & Lazenby (2019)	Developed own (USA-based but reflecting global faculty and institutional perspectives)(44)	Graduate global health nursing students	Discusses expectations for nurses who have completed graduate-level global health training	Pedagogy and assessment included use of case vignettes, essays and other types of critical analysis and reflection, development of plans and proposals, supervised clinical activities in host countries, discussion and reflection with host team members.

Target Audiences for Global Health Competencies

The scoping review identified three major groups of target audiences for public health and global health competencies, post-graduate public health students, professional development for global public health workers, and global health training for clinical and allied health professionals. The competencies for the first audience included U.S.-based training programs preparing individuals for public health careers internationally while the second category focuses more on training programs for LMIC based public health professionals. The last category includes individuals who may be from the US but ultimately who plan to work or already work in a low-resource health setting as a clinical or allied health professional.

Post-graduate Public Health Students: Two articles written by a team from the CUGH sub-committee on competencies broadly address global health professionals, recognizing the diversity of backgrounds and levels of expertise of public health and global health trainees.(13, 14) In Canada, multiple universities under the GHEC have engaged in individual as well as collective review and debate about global health competencies.(45) The ASPPH also reflects a university-based consensus-building process to determine competencies for post-graduate degree-seeking students across the Council on Education for Public Health (CEPH)-accredited U.S. universities.(35, 38)

Global Public Health Professionals: USAID and the Afya Bora Fellowship focus on professional skill-building targeted towards specific career trajectories, preparing leaders to manage global health programs in Africa.(33) The USAID competencies are geared towards employees within the USAID system and target political and managerial competencies as well as content areas aligned with global health-related USAID strategy.(18)

Clinical and Allied Health Professional Training Component: Nine articles focused on medical, nursing, pharmacy, dental, and rehabilitation students receiving targeted training in global health.(30, 46-50) For example, competencies based on the GHEC domains were adapted for family medicine trainees in Canada who plan to engage in global health; these include values and "soft skills" that will enable a physician to operate effectively and appropriately in diverse contexts.(48) A specialized matrix for global oral health was also developed building on the CUGH competency domains and resulted in a list focused on dental disease-specific knowledge, including disease risk factors and a set of more general interpersonal and professional skills.(49)

Defining Skill Levels and "Milestones" for Learners

As numerous groups continue to work on defining and refining competency domains, many have evolved to recommend specific levels or milestones for trainees, which can be tailored depending on career trajectory or scope of work.(45) Tools to organize and measure learner progress and growth within competency domains have begun to emerge, particularly in the last few years (2015-2019).(7, 23, 25, 51, 52) We identified two approaches to tracking the achievement of levels of competency across domains for professionals in public health and global health. The first approach focuses on sequential or tiered levels of ability and is more hierarchical in nature. This approach focuses more on the achievement of advancing or specialized skills. The second approach has a more longitudinal view and shows differing skills as the roles evolve (Figure 1). While the first approach is more likely to be tiered and perceived and implemented as a linear progression, the second can feasibly include parallel routes to different milestones and is more apt at placing individuals within a functional category.

Figure 1: Two Approaches identified for conceptualizing levels of competency

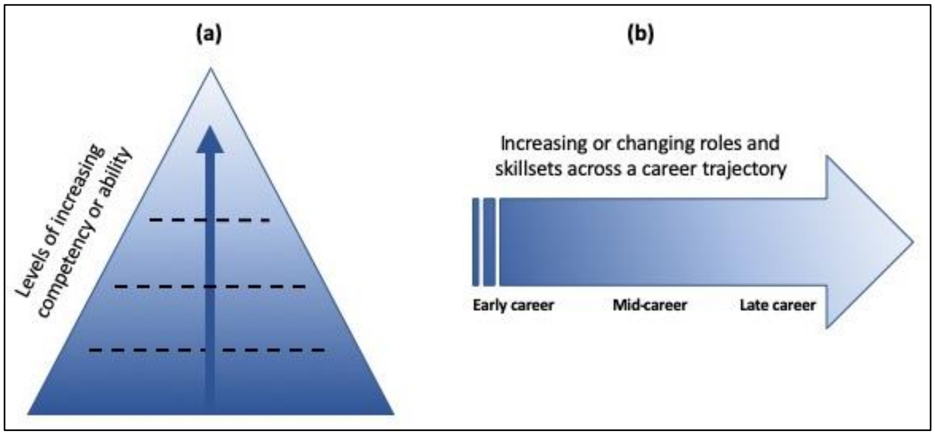

The USAID competency framework utilized a three-level approach with basic, intermediate, and advanced categories.(17, 18) Similarly, Gruppen, *et al.* (53) employed a four-level model with beginner levels focusing on knowledge, and more advanced levels focus on skills. The Afya Bora fellowship measures skills on a scale from weak to excellent.(33)

Building on CUGH's set of core competencies, a four-level approach was developed by CUGH beginning with a "Global Citizen" basic awareness level for a trainee pursuing a field with bearing on global health but not necessarily with sustained or direct engagement. It ends at an "Advanced" level of student who plans to have long-term engagement in global health and work towards holding substantive leadership positions.(14) The Global Emergency Medicine Think Tank Education Working Group (EWG) also used a structured process to determine milestones across five levels for emergency medicine trainees undertaking global health experiences. The levels ranged from Level 1 characterized by very basic knowledge of major concepts across competency domains to a Level 5, indicating a leader and teacher who is able to contribute to building the field of global health across one or more competency domains.(30)

Most of the clinically-oriented articles (except Douglass, et al.)(30) described specific milestones or expectations to be achieved by the end of the training programs, and several of the non-clinical examples (Sharma, et al., Winskell, et al., Hobson, et al., and the ASPPH)(34, 38, 54) also emphasized specific levels and milestones to be achieved by the completion of an MPH program.

Pedagogy and Assessment Strategies and Tools

Pedagogy: Pedagogical approaches revealed several consistent themes across the clinical and non-clinical articles. The first key theme was direct engagement and application of learning in clinical or field settings. For clinical settings, this could include practice with standardized patients, supervised clinical activities, other engagement with host team members, or similar apprenticeship models.(30, 44, 46, 48) For non-clinical field activities, these field placements included participation in activities from community diagnosis and program planning through to project evaluation.(38, 45, 55) Simulations for use in classroom settings were also a related theme found across both target audiences.(30, 48, 52, 53)

The second cross-cutting theme relates to group work. This theme included small group discussions in classroom settings, group projects, collaborative activities to develop proposals or other products, and group problem-solving in clinical contexts.(30, 33, 34, 38, 44, 48, 53) Eichbaum (22), in particular, noted that the role of group work may be seen differently across cultures that are more individualistic versus more collectivist, and also that learners may have different levels of experience, comfort, or expectations for how these group activities are managed. What is apparent is that while group work is identified as a core educational strategy, it may be more difficult to implement in certain cultural settings, and thus group work approaches that are facilitated or more structured may be important to adopt.

The final cross-cutting theme related to self-reflection, which was present for both clinical and non-clinical audiences as well as across levels of graduate and post-graduate training. Different articles mentioned different approaches, including reflective essays and journaling, as well as reflections with peers as a strategy to strengthen the absorption of curricula.(30, 38, 44, 45, 48, 56)

Assessment Strategies and Tools: We identified three kinds of evaluation tools that have been developed and used to assess individual competencies: i) self-assessment surveys, ii) assessment from multiple stakeholders or perspectives, and iii) mixed methods assessments using surveys, scenarios or interviews. The findings for each kind will be discussed in sequence.

Several of the articles included self-reflection approaches as a means to facilitate assessment. The Afya Bora fellowship was the only group that utilized a self-reflection tool both before and after each module of the curriculum to demonstrate a change in knowledge/skills/comfort. .(33) Veras, *et al*. (2012) published the results of a validated self-assessment survey developed to assess global health competencies (57) and also utilized the tool with rehabilitation students to self-assess knowledge, skills, as well as learning needs.(40) The tool begins to address the need for validated structured assessment that is able to assess gaps knowledge across different participant groups uniformly and has since been applied to global health training in South Korea with positive results as well as with rehabilitation students in Canada.(40, 46, 57) Gladding, *et al.*(56) and Redwood-Campbell, *et al.*(48) also emphasized the need to better evaluate student self-reflection and essays to assess progress towards milestones or other training-specific goals.

Two documents included in the scoping review utilized input from multiple perspectives, including self-evaluation.(25, 58) Both used a variation of a 360 evaluation approach that

included feedback from the public health professional's subordinates, colleagues, and supervisors, as well as a self-evaluation by the individual themselves. Both approaches utilized Likert scales, though one scale focused on skill development, and the other focused on the development of competency in a particular area from the role of learner to that of teacher/instructor. USAID also emphasized feedback from supervisors and other performance reviews (18), and Eichbaum (22) advocated for evaluation from multiple sources, including faculty, representatives from the health system, and self-evaluation.

Wroe, *et al.* (2017) developed a series of scenarios common to global health practice in order to capture more nuanced feedback related to the "soft skills" in global health. Interviewers assessed whether respondents had received adequate global health training in order to be prepared for continued practice in areas such as professionalism, self-care, and interpersonal and cross-cultural communication.(52) Another approach by Knight, *et al.* (55) utilized a self-assessment survey that included quantitative measures as well as open-ended qualitative questions.

Douglass and colleagues (2017) hypothesized that the evaluation strategy might need to be responsive to the level of the learner, and thus, the skill being assessed (30). While earlier levels focus more on self-reflection and formal assessment processes, the more advanced levels require more objective measures of achievement, such as peer assessment or scientific publications. (30)

Discussion

The implementation of competency-based educational models is both nuanced and challenging. A variety of approaches have been adopted for different learner groups. However, most focus on developing specific measures for achievement, such as requirements for

completing a degree program, and use a variety of evaluation strategies. The challenge of a meaningful and appropriate assessment of competence (59) has led to efforts to offer more concrete approaches through the development of levels, milestones, and evaluation tools.

Much of the public health and global health thinking has been built on the foundation of competency-based medical education (CBME) from clinical settings.(60) Many of the best practices have developed specific anchors or skills that can be objectively observed or otherwise identified to define the achievement of a competency level. It is however, acknowledged that challenges remain on how to meaningfully assess “soft skills”, such as leadership, communication, and cross-cultural practice, which are central to global public health work.(61) Assessment of “soft-skills” may require a process-oriented approach to understand learner experiences and establish feedback mechanisms. These competencies ultimately also need to be developed at an individual, program, and broader agency or societal levels and involve a variety of stakeholders (educators, peers, supervisors) within training programs as well as post-graduation.(62)

Another promising direction is the emerging focus on adapting competencies for use in LMIC. (26, 46, 58) This has included assessing competence in LMICs while accounting for local learning styles, culture, and other contextual factors relevant to global health work. However challenges do exist, for example, assessment of competencies within cultures where teamwork and direct engagement are commonplace and necessary but traditional assessment approaches do not capture these skills fully (22, 50) or how to account for cultural and practice setting differences when aiming to develop a globally-applicable set of competencies.(50) In the field of global public health, increased emphasis is needed on the competencies related to participatory

approaches, learning across disciplines and in resourceful ways, and maintaining a social justice and health equity lens.(22)

Long-term capacity strengthening in LMICs to achieve the Sustainable Development Goals (SDGs) must consider the context in which global health practice occurs.(63, 64) Further evidence on approaches used in LMIC settings and their effectiveness is needed. Future efforts might include systematically documenting consensus on competencies based on empirical studies that include input from a wide range of global public health stakeholders, including LMIC national-level policymakers and leaders, managers, academics and researchers, and civil society. Competency-based education is held up as an approach that can make training as applied and impactful as possible. However, further rigorous evaluation of the impact—both immediate and longer-term—of global health training programs is needed. Furthermore, competency-based education for students and early-career professionals undergoing more knowledge-focused training is inherently different from the skills desired among senior health professionals. Colloquially these skills are often termed "leadership skills." Understanding the nuances of how leadership skills can strengthen public health practice and how these skills and be codified to provide focused monitoring, feedback, and training is the key to supporting the global health workforce.

Our review had several limitations, which included reliance on published literature and ongoing ambiguity around the most appropriate search terms to utilize. To the first point, although we have included published and grey literature in this review, we believe that there are more examples of implementation of competency-based curricula in LMIC contexts that what we were able to find. This links with the second point, which is that different programs, educational systems, and health professions use a range of different terms to describe both global public

health as well as competency-based curricula. Therefore, conducting a systematic and exhaustive search was a challenge.

Conclusions

Global public health is already a very dynamic field and is sure to change even more in the future. While many different voices are joining the debate about how the field will evolve by providing perspectives, tools, and learning activities, a great deal of work must be done to align, validate and evolve these contributions towards translatable, actionable, and trustworthy instruments, resources, and opportunities. The capacity development needs of professionals in government versus non-governmental organizations, academic or research- versus program implementation-focused institutions, and the public or non-profit versus private sectors can vary greatly, as can the individual learning styles of professionals in those settings. Competencies and their assessment may also need to vary accordingly to respond to the needs of specific agencies or particular groups of learners. Further discussion and action on the role and implementation of competency-based education better equip the global public health workforce as they address current and emerging global health challenges is needed.

Acknowledgments: This work is supported by The Sustaining Technical and Analytic Resources (STAR) project. STAR is a five-year cooperative agreement funded by the United States Agency for International Development Global Health Bureau (USAID/GHB), beginning May 1, 2018. It is implemented by the Public Health Institute, in collaboration with Johns Hopkins University, University of California, San Francisco, and the Consortium of Universities for Global Health. Contents are the responsibility of the STAR project, Johns Hopkins

University, and the Public Health Institute, and do not necessarily reflect the views of USAID or the US Government.

References

1. Koplan JP, Bond TC, Merson MH, Reddy KS, Rodriguez MH, Sewankambo NK, et al. Towards a common definition of global health. Lancet. 2009;373(9679):1993-5.
2. Buse K, Hawkes S. Health in the sustainable development goals: ready for a paradigm shift? Global Health. 2015;11:13.
3. Pruitt SD, Epping-Jordan J. Preparing the 21st Century Global Healthcare Workforce. British Medical Journal. 2005;330:637-9.
4. Chen L, Evans T, Anand S, Boufford JI, Brown H, Chowdhury M, et al. Human Resources for Health: Overcoming the Crisis. The Lancet. 2004;364(9449):1984-90.
5. Gupta R, Bush BP, Dorsey J, Moore E, van der Hoof Holstein C, Farmer PE. Improving the global health workforce crisis: an evaluation of Global Health Corps. Lancet Glob Health. 2015;3(11):e679.
6. Rudy S, Wanchek N, Godsted D, Blackburn M, Mann E. The PHI/GHFP-II Employers' Study: The Hidden Barriers Between Domestic and Global Health Careers and Crucial Competencies for Success. Ann Glob Health. 2016;82(6):1001-9.
7. Sawleshwarkar S, Negin J. A Review of Global Health Competencies for Postgraduate Public Health Education. Front Public Health. 2017;5:46.
8. Roegiers X. A Conceptual Framework for Competencies Assessment. Current and Critical Issues in the Curriculum and Learning: International Bureau of Education (IBE) and UNESCO; 2016.
9. Gaudet C, Annulis HM, Kmiec JJ. Building an Evaluation Framework for a Competency-based Graduate Program at the University of Southern Mississippi. Performance Improvement. 2008;47(1).
10. ASTD. ASTD Competency Model. American Society for Training and Development (ASTD); 2019.
11. SRHM. Society for Human Resource Management (SRHM) Competency Model. Society for Human Resource Management (SRHM); 2012.
12. ASPH. Master's Degree in Public Health Core Competency Development Project. 2006.
13. Wilson L, Callender B, Hall TL, Jogerst K, Torres H, Velji A. Identifying global health competencies to prepare 21st century global health professionals: report from the global health competency subcommittee of the consortium of universities for global health. J Law Med Ethics. 2014;42 Suppl 2:26-31.
14. Jogerst K, Callender B, Adams V, Evert J, Fields E, Hall T, et al. Identifying interprofessional global health competencies for 21st-century health professionals. Ann Glob Health. 2015;81(2):239-47.
15. Arthur MA, Battat R, Brewer TF. Teaching the basics: core competencies in global health. Infect Dis Clin North Am. 2011;25(2):347-58.
16. Ablah E, Biberman DA, Weist EM, Buekens P, Bentley ME, Burke D, et al. Improving global health education: development of a Global Health Competency Model. Am J Trop Med Hyg. 2014;90(3):560-5.
17. USAID. Foreign Service Skills Framework. Washington, DC: USAID; 2016.
18. USAID. Service, Foreign Services, and Foreign . Services National Competency and Proficiency Catalog : Human Capital and Talent Management (HCTM). Washington, DC2017.
19. Public Health Foundation. Core Competencies for Public Health Professionals. 2014.
20. Public Health Foundation. Competency Assessment: Tier 3 Public Health Processionals. Washington, DC: Public Health Foundation and University of North Carolina; 2014.
21. Calhoun JG, Spencer HC, Buekens P. Competencies for global heath graduate education. Infect Dis Clin North Am. 2011;25(3):575-92, viii.
22. Eichbaum Q. Acquired and Participatory Competencies in Health Professions Education: Definition and Assessment in Global Health. Acad Med. 2017;92(4):468-74.
23. Walpole SC, Shortall C, van Schalkwyk MC, Merriel A, Ellis J, Obolensky L, et al. Time to go global: a consultation on global health competencies for postgraduate doctors. Int Health. 2016;8(5):317-23.
24. Bartee T, Winnail S, Olsen S, Diaz C, Blevens JA. Assessing Competencies of a Public Health Workforce in a Frontier State. Journal of Community Health. 2003;28(6):459-69.

25. Issel LM, Baldwin KA, Lyons RL, Madamala K. Self-reported competency of public health nurses and faculty in Illinois. Public Health Nurs. 2006;23(2):168-77.
26. Zwanikken PA, Alexander L, Huong NT, Qian X, Valladares LM, Mohamed NA, et al. Validation of public health competencies and impact variables for low- and middle-income countries. BMC Public Health. 2014;14:55.
27. Drain PK, Holmes KK, Skeff KM, Hall TL, Gardner P. Global health training and international clinical rotations during residency: current status, needs, and opportunities. Acad Med. 2009;84(3):320-5.
28. Frenk J, Chen L, Bhutta Z, Cohen J, Crisp N, Evans T, et al. Health Professionals for a New Century: Transforming Education to Strengthen Health Systems in an Interdependent World. Lancet. 2010;376:1923-58.
29. Kerry VB, Ndung'u T, Walensky RP, Lee PT, Kayanja VF, Bangsberg DR. Managing the demand for global health education. PLoS Med. 2011;8(11):e1001118.
30. Douglass K, Jaquet G, Hayward A, Dreifuss B, Tupesis J. Development of a Global Health Milestones Tool for Learners in Emergency Medicine: A Pilot Project. Society for Academic Emergency Medicine. 2017.
31. Arksey H, O'Malley L. Scoping Studies: Towards a Methodological Framework. International Journal of Social Research Methodology. 2005;8(1).
32. CCGHR. Canadian Coalition for Global Health Research. 2019.
33. Afya Bora Fellowship,. 2019.
34. Winskell K, Evans D, Stephenson R, Rio CD, Curran J. Incorporating Global Health Competencies into the Public Health Curriculum. ASPPH From the Schools and Programs of Public Health. 2014;129:203-9.
35. ASPH Education Committee. Master's Degree in Public health Core Competency Development Project Version 2.3. Atlanta, GA2016.
36. Jardine D, Deslauriers J, Kamran S, Khan N, Hanstra S, Edgar L. Milestones Guidebook for Residents and Fellows. ACGME; 2017.
37. Hamer D, Hansoti B, Prabhakaran D, Huffman M, Nxumalo N, Fox M, et al. Global Health Research Mentorship Competencies for Individuals and Institutions in Low- and Middle-Income Countries. American Journal of Tropical Medicine and Hygiene. 2019;100:15-9.
38. Hobson K, Coryn C, Fierro L, Sherwood-Laughlin C. Instruction of Evaluation Competencies in Council on Education for Public Health (CEPH)-Accredited Master of Public Health (MPH) Degree Programs. American Journal of Evaluation. 2019.
39. CanMEDS. Canadian Medical Education Directives for Specialists (CanMEDS). 2019.
40. Veras M, Pottie K, Cameron D, Dahal G, Welch V, Ramsay T, et al. Assessing and Comparing Global Health Competencies in Rehabilitation Students. Rehabilitation Research and Practice. 2013.
41. WHO. Towards better leadership and management in health. Making Health Systems Work Series, Working Paper No 10. Geneva, Switzerland: World Health Organization; 2007.
42. Ried U, Weller B. Nursing human resources planning and management competencies. Geneva, Switzerland: International Council of Nurses (ICN); 2010.
43. Health Professions Council of South Africa. Medical and dental professions board: core competencies for undergraduate students in clinical associate, dentistry and medical teaching and learning programmes in South Africa. Pretoria, South Africa: Health Professions Council of South Africa (HPCSA); 2014.
44. Kelly T, Lazenby M. Developing and validating learning domains, competencies, and evaluation items for global health clinical immersion practicums for graduate-level nursing programs. J Adv Nurs. 2019;75(1):234-52.
45. Cole DC, Davison C, Hanson L, Jackson SF, Page A, Lencuch R, et al. Being global in public health practice and research: complementary competencies are needed. Can J Public Health. 2011;102(5):394-7.

46. Kim Y, Han K, Yoo HY. Enhancing undergraduate nursing students' global health competencies in South Korea. Public Health Nurs. 2017;34(5):479-84.
47. Battat R, Seidman G, Chadi N, Chanda MY, Nehme J, Hulme J, et al. Global health competencies and approaches in medical education: a literature review. BMC Med Educ. 2010;10:94.
48. Redwood-Campbell L, Pakes B, Rouleau K, MacDonald CJ, Ayra N, Pukey E, et al. Developing a Curriculum Framework for Global Health in Family Medicine: Emerging Principles, Competencies, and Educational Approaches. BMC Medical Education. 2011;11(46):1-8.
49. Benzian H, Greenspan J, Barrow J, Hutter J, Loomer P, Stauf N, et al. A Competency Matrix for Global Oral Health. Global Dental Education. 2015:353-62.
50. Bruno A, Bates I, Brock T, Anderson C. Towards a global competency framework. Am J Pharm Educ. 2010;74(3):56.
51. Veras M, Pottie K, Welch V, Labonte R, Eslava-Schmalbach J, Borkhoff CM, et al. Reliability and validity of a new survey to assess global health competencies of health professionals. Glob J Health Sci. 2013;5(1):13-27.
52. Wroe EB, McBain RK, Michaelis A, Dunbar EL, Hirschhorn LR, Cancedda C. A Novel Scenario-Based Interview Tool to Evaluate Nontechnical Skills and Competencies in Global Health Delivery. J Grad Med Educ. 2017;9(4):467-72.
53. Gruppen LD, Mangrulkar RS, Kolars JC. The promise of competency-based education in the health professions for improving global health. Hum Resour Health. 2012;10:43.
54. Sharma K, Zodpey S, Morgan A, Gaidhane A, Syed ZQ, Kumar R. Designing the framework for competency-based master of public health programs in India. J Public Health Manag Pract. 2013;19(1):30-9.
55. Knight S, Ross A, Mahomed O. Developing Primary Health Care and Public Health Competencies in Undergraduate Medical Students. South African Family Medicine. 2017;59(3):103-9.
56. Gladding S, Zink T, Howard C, Campagna A, Slusher T, John C. International electives at the university of Minnesota global pediatric residency program: opportunities for education in all Accreditation Council for Graduate Medical Education competencies. Acad Pediatr. 2012;12(3):245-50.
57. Veras M, Pottie K, Welch V, Labonte R, Eslava-Schmalbach J, Borkhoff CM, et al. Reliability and validity of a new survey to assess global health competencies of health professionals. Glob J Health Sci. 2012;5(1):13-27.
58. Munyewende PO, Levin J, Rispel LC. An evaluation of the competencies of primary health care clinic nursing managers in two South African provinces. Glob Health Action. 2016;9:32486.
59. Eichbaum Q. The problem with competencies in global health education. Acad Med. 2015;90(4):414-7.
60. Epstein RM, Hundert EM. Defining and assessing professional competence. JAMA. 2002;287(2):226-35.
61. Allegrante JP, Barry MM, Airhihenbuwa CO, Auld ME, Collins JL, Lamarre MC, et al. Domains of core competency, standards, and quality assurance for building global capacity in health promotion: the galway consensus conference statement. Health Educ Behav. 2009;36(3):476-82.
62. Holmboe ES, Sherbino J, Long DM, Swing SR, Frank JR. The role of assessment in competency-based medical education. Med Teach. 2010;32(8):676-82.
63. Crump JA, Sugarman J, (WEIGHT) WGoEGfGHT. Ethics and best practice guidelines for training experiences in global health. Am J Trop Med Hyg. 2010;83(6):1178-82.
64. Thistlethwaite J. Interprofessional education: a review of context, learning and the research agenda. Med Educ. 2012;46(1):58-70.

Figure

Click here to access/download;Figure;Figure 1 GH comps milestones paper final 12_2019.tiff

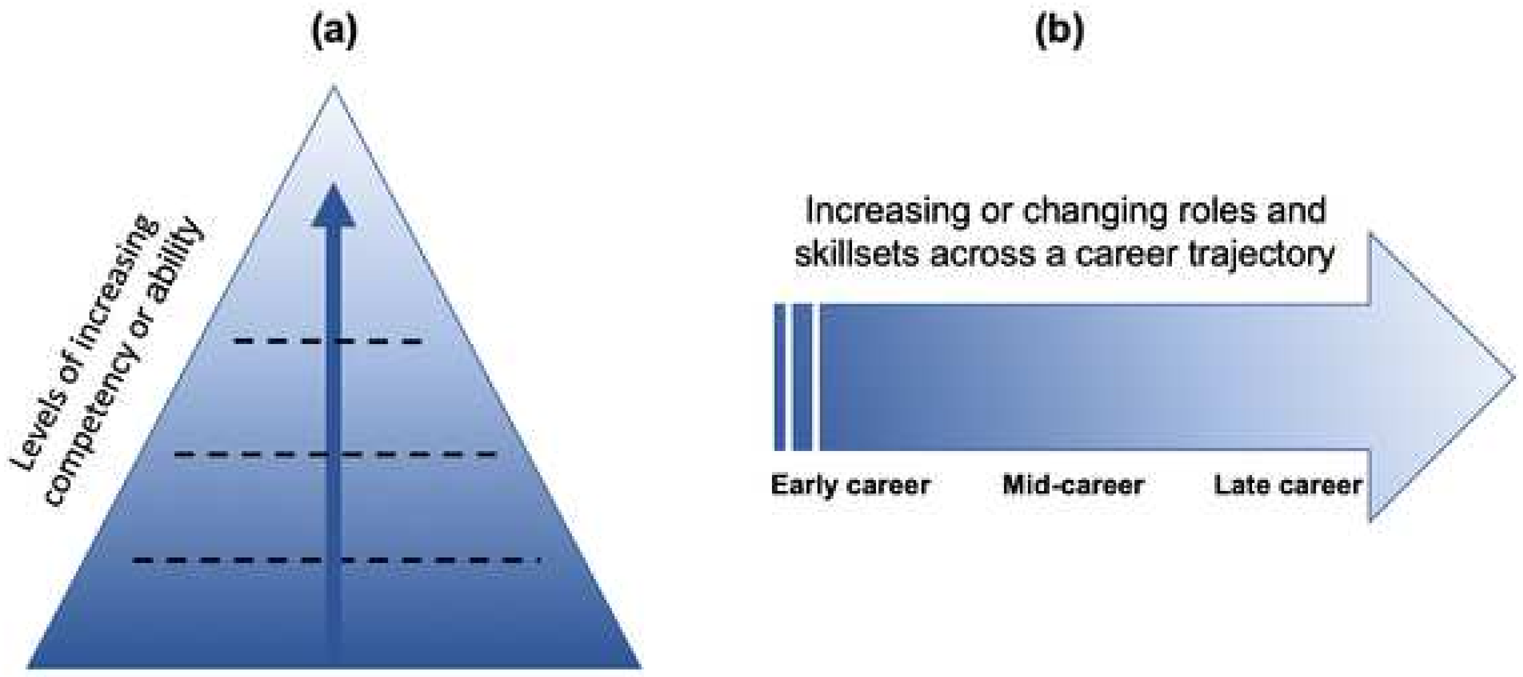

Click here to access/download
Supporting Information
Appendix 1 Search Strategy.docx

Click here to access/download
Supporting Information
Schleiff et al PRISMA-ScR-Fillable-Checklist-1_BH.docx
